# Bacterial communities in the solid, liquid, dorsal, and ventral epithelium fractions of yak (*Bos grunniens*) rumen

**DOI:** 10.1002/mbo3.963

**Published:** 2019-11-07

**Authors:** Qingmiao Ren, Huazhe Si, Xiaoting Yan, Chang Liu, Luming Ding, Ruijun Long, Zhipeng Li, Qiang Qiu

**Affiliations:** ^1^ State Key Laboratory of Grassland Agro‐Ecosystems School of Life Sciences Lanzhou University Lanzhou China; ^2^ Department of Special Animal Nutrition and Feed Science Institute of Special Animal and Plant Sciences Chinese Academy of Agricultural Sciences Changchun China; ^3^ Research Center for Ecology and Environmental Sciences Northwestern Polytechnical University Xi’an China

**Keywords:** *Bos grunniens*, *Campylobacter* spp., dorsal and ventral epithelium, ecology niches, *Howardella* spp, rumen, solid and liquid

## Abstract

Yak (*Bos grunniens*) is an important and dominant livestock species in the challenging environment of the Qinghai–Tibetan Plateau. Rumen microbiota of the solid, liquid, and epithelium fractions play key roles in nutrient metabolism and contribute to host adaptation in ruminants. However, there is a little knowledge of the microbiota in these rumen fractions of yak. Therefore, we collected samples of solid, liquid, dorsal, and ventral epithelium fractions from five female yaks, then amplified bacterial 16S rRNA gene V4 regions and sequenced them using an Illumina MiSeq platform. Principal coordinates analysis detected significant differences in bacterial communities between the liquid, solid, and epithelium fractions, and between dorsal and ventral epithelium fractions. *Rikenellaceae* RC9, the families *Lachnospiraceae* and *Ruminococcaceae*, and *Fibrobacter* spp. were the abundant and enriched bacteria in solid fraction, while the genera *Prevotella* and *Prevotellaceae* UCG 003 were higher in the liquid fraction. *Campylobacter* spp., *Comamonas* spp., *Desulfovibrio* spp., and *Solobacterium* spp. were significantly higher in dorsal epithelium, while *Howardella* spp., *Prevotellaceae* UCG 001, *Ruminococcaceae* UCG 005, and *Treponema* 2 were enriched in the ventral epithelium. Comparison of predictive functional profiles among the solid, liquid, and dorsal, and ventral epithelium fractions also revealed significant differences. Microbiota in the ventral fraction of yak rumen also significantly differ from reported microbiota of cattle. In conclusion, our results improve our knowledge of the taxonomic composition and roles of yak rumen microbiota.

## Graphical abstract

In this present study, we explored microbiota in the solid, liquid, and epithelium (dorsal and ventral) fractions of yak rumen, and compared functional profiles of microbiota of the four fractions, and also compared microbiota of the ventral epithelium between yak and cattle. Our results indicated the significant differences in bacterial communities between the liquid, solid, and epithelium fractions, and between dorsal and ventral epithelium fractions. Moreover, comparison of functional profiles among the solid, liquid, and dorsal and ventral epithelium fractions also revealed significant differences. Finally, we found that the microbial composition and functional profile in the ventral epithelium between yak and cattle significantly differed, suggesting the specific role of microbiota in the ventral epithelium of yak rumen.

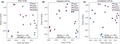

## 1 INTRODUCTION

The yak (*Bos grunniens*) is a remarkable domesticated ruminant species of the Qinghai–Tibetan Plateau (QTP), which provides basic necessities for Tibetans including meat, milk, and transportation (Wiener, Han, & Long, 2003). However, yak is seriously challenged by the harsh environmental conditions, such as high altitude (>3,000 m), low temperatures, and oxygen tension (Zhou, Zhong, et al., 2017a; Zhou, Fang, et al., 2017b), and limitations in both quality and availability of food associated with the harsh conditions and short growing season (mid‐May to mid‐September; Wang et al., 2011). Hence, yaks have evolved unique genomic features and associated traits that enable them to survive in this environment. For instance, significant enrichment or expansion of genes involved in sensory perception, hypoxia tolerance, and nutrient metabolism has been found in the yak genome, relative to the genome of closely related low‐altitude cattle (Qiu et al., 2012). Previous studies have also shown that yaks have lower daily fasting nitrogen excretion rates (Wang et al., 2011) and higher nitrogen retention than cattle (Wang et al., 2011), suggesting that they have higher dietary efficiency. Moreover, recent studies have demonstrated that methanogen communities in yak and cattle rumen significantly differ (Huang, Tan, Long, Liang, & Wright, 2012), and the yak rumen microbiome is significantly enriched with genes linked to pathways yielding volatile fatty acids (VFAs), and genes associated with VFA transport and absorption in the ruminal epithelium are significantly upregulated (Zhang et al., 2016). Together, these results indicate that the rumen microbiota are highly important in yak nutrient metabolism and adaptation.

The complex rumen microbial ecosystem can be divided into three: solid, liquid, and epithelial fractions (Cho et al., 2006; De Mulder et al., 2017; Liu, Zhang, Zhang, Zhu, & Mao, 2016; Sadet, Martin, Meunier, & Morgavi, 2007; Schären et al., 2017). The solid microbiota attached to ingested plant material play a key role in fiber digestion (McAllister, Bae, Jones, & Cheng, 1994), while the liquid phase contains bacteria that strongly participate in metabolism of soluble nutrients, and transmits components of the solid‐adherent biofilms to newly ingested feed (De Mulder et al., 2017). There are also significant differences between the microbial communities in the solid and liquid fractions (De Mulder et al., 2017; Larue, Yu, Parisi, Egan, & Morrison, 2005; McAllister et al., 1994). Microbiota of the epithelial fraction also have distinct functions, particularly oxygen scavenging (Cheng, Mccowan, & Costerton, 1979), urea hydrolysis (Cheng & Wallace, 1979), and recycling of epithelial tissue (Dinsdale, Cheng, Wallace, & Goodlad, 1980). These previous findings clearly suggest that fractions of rumen microbiota may generally have distinct compositional and functional signatures. However, although the microbiota in the yak rumen and other stomach components have been examined (Guo et al., 2015; Huang et al., 2012; Xue et al., 2016, 2018; Zhou, Zhong, et al., 2017a; Zhou, Fang, et al., 2017b), we have a little knowledge of the composition of microbiota of the three fractions in the yak rumen and differences between them.

The rumen epithelium can also be divided into dorsal and ventral fractions. Previous studies demonstrated that the dorsal epithelium faction faces an accumulation of gas dome, while the ventral epithelium fraction meets to a relatively fluid digest in the rumen (Tafaj et al., 2004). The degree of papillation also significantly differs between dorsal and ventral regions (Clauss, Hofmann, Fickel, Streich, & Hummel, 2009). Moreover, yak produces significantly lower levels of methane and higher levels of VFAs than cattle, and rumen microbiomes of yak and cattle differ (Zhang et al., 2016). Therefore, comparing the ventral epithelium microbiota in yak and cattle could enhance understanding of roles of microbiota in adaptations of yak and other ruminants.

Thus, aims of this study were to characterize and compare microbiota in the solid, liquid, and epithelium (dorsal and ventral) fractions of yak rumen; to compare functional profiles of microbiota of the four fractions; and to compare microbiota of the ventral epithelium in yak and cattle.

## 2 MATERIAL AND METHODS

### 2.1 Animals and sampling

In this study, five female 4‐year‐old yaks in Hezuo city, Gansu province, China (latitude >3,000 m) were used. The yaks were raised by the local herdsman, who traditionally grazes yaks on natural alpine meadow grassland (mainly consisted of *Kobresia* spp. and *Cyperaceae* spp.) without feed supplements, from September to November 2018. On the morning, the yaks were fed the collected pasture from the same grassland, which were then slaughtered after 3 hours of feeding. To minimize potential contamination from other gut regions, each yak carcass was placed in a natural position and the rumen chambers were tied off using cotton rope. Then, each animal's rumen content was carefully collected and homogenized. Approximately 200 g portions of whole contents were taken by hands with sterile gloves and squeezed through four layers of cheesecloth to separate them into solid and liquid fractions. Finally, the solid and liquid samples obtained were stored in DNase‐ and RNase‐free tubes. In addition, dorsal and ventral epithelium samples were collected by cutting ca. 3 cm^2^ pieces of epithelial tissue from dorsal and ventral rumen, then washing them with cold 0.01 M phosphate‐buffered saline three times. After that, rumen epithelium‐associated microbiota samples were scraped off using sterilized glass slides. All samples were immediately placed in liquid nitrogen and stored at −80°C for further analysis.

### 2.2 DNA extraction, library construction, and next‐generation sequencing

The microbial genomic DNA in each rumen sample was extracted following published methods (Yu & Morrison, 2004). Its integrity and quantity was evaluated by 1.0% agarose gel electrophoresis and spectroscopic analysis with a ND‐1000 spectrometer (NanoDrop). The V4 region of bacterial 16S rRNA genes in each sample was amplified in triplicate (Klindworth et al., 2012). Amplicons were purified using an AxyPrep DNA Gel Extraction Kit (Axygen Biosciences) according to the manufacturer's instructions. Purified PCR products were quantified using a Qubit^®^3.0 fluorometer (Invitrogen). A MiSeq Reagent Kit v2 was then used to construct an Illumina Pair‐End library according to the manufacturer's instructions. Finally, the obtained amplicons were sequenced using an Illumina MiSeq platform to generate paired 250‐bp reads.

### 2.3 Bioinformatics and statistical analysis

To compare the epithelial microbiota in yak and cattle, sequences from cattle obtained in a previous study, using the same primers (De Mulder et al., 2017), were downloaded. The paired end sequences were first assembled into contigs using FLASH (Magoč & Salzberg, 2011) with truncation of reads at any site receiving an average quality score <20 over a 50‐bp sliding window, removal of reads shorter than 50 bp after truncation, no mismatches in primers, and merger of sequences with >10 bp overlaps. Then, all sequences were analyzed using QIIME 1.9.1 (Caporaso et al., 2010). The operational taxonomy units (OTUs) were clustered using UPARSE based on 97% sequence similarity (Edgar, 2013). Chimeric sequences were identified and removed using UCHIME (Edgar, Haas, Clemente, Quince, & Knight, 2011). Representative sequences of the OTUs were used for taxonomic classification in conjunction with the SILVA database (version 123; Quast et al., 2013; Wang, Garrity, Tiedje, & Cole, 2007). Representative sequences in each OTU were also aligned using MUSCLE (Edgar, 2004), then used to construct a phylogenetic tree with FastTree (Price, Dehal, & Arkin, 2009). Singletons were removed, and the sequences from each sample were subsampled to the minimum number (36,574) in order to decrease the effects of sequencing depth. The alpha diversity indices including Chao1 and Shannon were also calculated using QIIME 1.9.1 (Caporaso et al., 2010).

Principal coordinate analysis (PCoA) was applied to compare the bacterial communities in the solid, liquid, dorsal, and ventral fractions based on Bray–Curtis, unweighted UniFrac and weighted UniFrac distances. Analysis of similarities (ANOSIM) was applied to test whether the microbial communities among these fractions are significantly different, and Adonis was employed to describe the strength and significance between them. In addition, the linear discriminant analysis (LDA) effect size (LEfSe) was used to identify the enriched bacterial taxonomy from each fraction (Segata et al., 2011). LEfSe first applies the nonparametric factorial Kruskal–Wallis rank‐sum test to detect significantly differing features (here, taxa), and then LDA to estimate the effect size of each feature. A significance level of *p* < .05 and effect‐size threshold of 3 were applied in this study to identify significant taxa. Finally, phylogenetic investigation of communities by reconstruction of unobserved states (PICRUSt) was used to predict functional profiles of the four fractions resulting from reference‐based OTU picking against the Greengenes database (Langille et al., 2013). The predicted genes were then clustered and categorized according to Kyoto Encyclopedia of Genes and Genomes (KEGG) pathways.

Differences in alpha diversity indices and functional category abundances among the four fractions were analyzed using one way ANOVA with the post hoc Tukey‐HSD test. All *p*‐values were corrected to account for the false discovery rate (FDR) by the Benjamini–Hochberg method, and FDR‐corrected *p*‐values below .05 were considered as significance. All values reported here are expressed as mean ± standard deviation (*SD*) unless otherwise stated.

### 2.4 Quantitative real‐time PCR for bacterial community

The bacterial 16S rRNA gene copy number was determined by quantitative PCR on a ABI 7300 real‐time PCR System (Life Technologies) suing SYBR Premix Ex Taq dye (TaKaRa Biotechnology). The primers reported in previous study were used for the quantitative PCR (Maeda et al., 2003). Each 20 µl reaction mixture contained 10 µl SYBR *Premix Ex* Taq^™^ (TaKaRa Biotechnology), 0.4 µl of each primer (10 µM), 0.4 µl ROX Reference Dye (TaKaRa Biotechnology), 6.8 µl of nuclease‐free water, and 2 μl of the template. The PCR was performed in a two‐step thermal cycling process that consisted of hot start activation at 95°C for 30 s, followed by 40 cycles at 95°C for 5 s, and 60°C for 1 min. The quantification of bacterial 16S rRNA gene copies in each sample was performed in triplicate, and the mean value was calculated. A standard curve was prepared by using a 10‐fold serial dilution of purified plasmid DNA containing the 16S rRNA gene sequence. The total number of gene copies was expressed as log10 numbers of marker loci gene copies per ng DNA.

## 3 RESULTS

### 3.1 16S rRNA gene sequencing and bacterial diversity in the rumen of yak

From the sequencing of bacterial 16S rRNA genes in the solid, liquid, dorsal, and ventral epithelial fractions of yak rumen, we obtained 1,009,806 raw sequence reads in total, with 50,490 ± 6,620 from each sample on average. After quality control, a total of 957,559 high‐quality sequences were retained, with 47,877 ± 6,443 on average (range: 36,574–60,816) per sample. As already mentioned, to minimize the sequencing depth effect, we subsampled sequences from each sample to the minimum number (36,574) and identified 4,200 OTUs in total in all samples based on 97% similarity.

**Figure 1:**
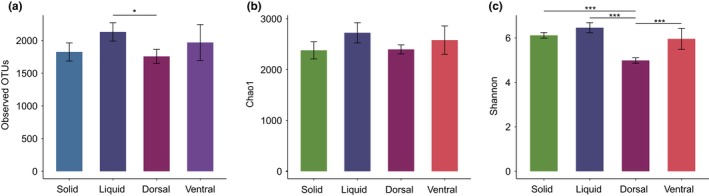
Alpha diversity indices of the solid, liquid, and epithelial fractions of yak rumen. *and ***indicate *p* < .05 and *p* < .001, respectively

All three indices (number of OTUs, Shannon and Chao 1) indicated that bacterial diversity was highest and lowest in the liquid and dorsal epithelium fractions, respectively (Figure 1). The Chao 1 index did not significantly differ among the four fractions (*p* > .05, Figure 1). However, the number of OTUs and Shannon diversity did significantly differ between them (*p* < .05, Figure 1).

### 3.2 Bacterial community composition in the rumen solid, liquid, and epithelial fractions of yak

**Figure 2:**
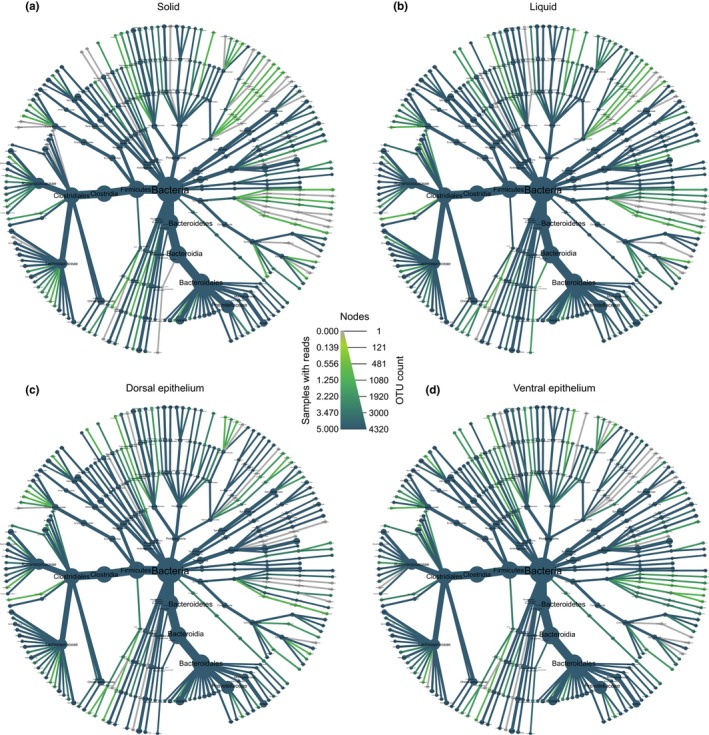
Heat trees showing the bacterial community composition in the (a) solid, (b) liquid, (c) dorsal epithelium, and (d) ventral epithelium fractions of yak rumen. Sizes of nodes and edges indicate OTU richness, and color indicates abundance

**Figure A1:**
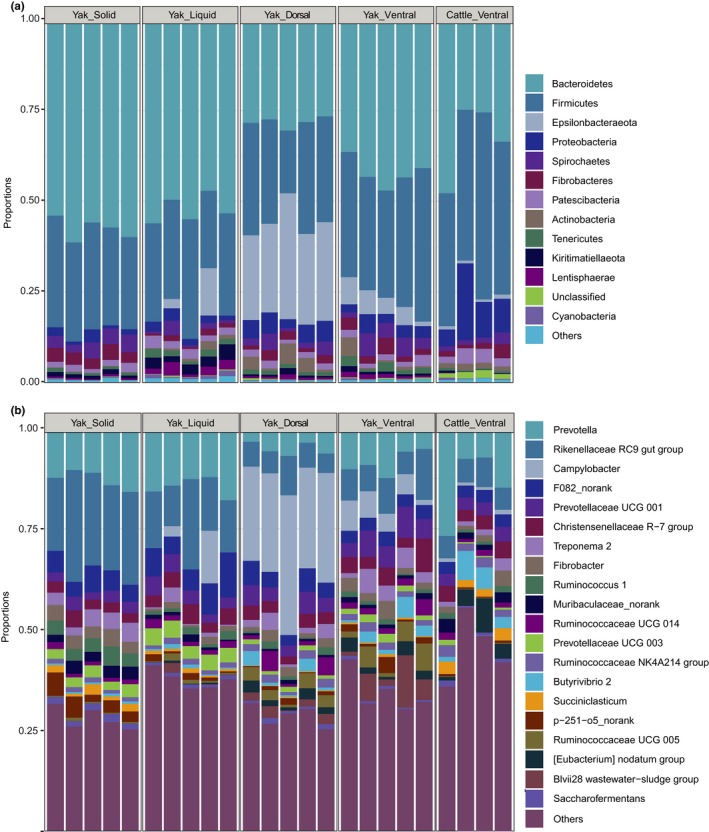
Stacked bar plot presenting the phylum composition in solid, liquid, dorsal epithelium and ventral epithelium fractions of yak and ventral epithelium fraction of cattle [Correction added on 6 January 2020 after first online publication: Figure A1 from Supporting Information has been moved to Appendix section]

Overall, a total of 19 phyla, 37 classes, 75 orders, 121 families, and 287 genera were identified in the four yak rumen fractions (Figure 2 and Figure A1 in Appendix). At the phylum level, the most abundant bacteria, accounting for more than 76% of the bacterial community in total were *Bacteroidetes* (57.6 ± 2.9, 52.2 ± 3.7 and 42.2 ± 3.9% in solid, liquid, and ventral epithelium, respectively) and *Firmicutes* (27.9 ± 2.1, 27.3 ± 4.1 and 34.6 ± 5.7% in solid, liquid, and ventral epithelium, respectively). However, in the dorsal epithelium fraction, the most abundant phylum was *Bacteroidetes* (28.3 ± 1.5%), followed by *Firmicutes* (27.4 ± 5.7%) and *Epsilonbacteraeota* (26.9 ± 4.5%).

At genus level, the most abundant bacteria in the solid, liquid, and ventral epithelium fractions were members of the *Rikenellaceae* RC9 gut group (22.5 ± 3.5, 14.5 ± 4.6, and 8.3 ± 2.7%, respectively) and *Prevotella* (12.8 ± 2.2%, 14.6 ± 2.3, and 8.6 ± 3.0%, respectively; Figure 2 and Figure A1 in Appendix). However, the most abundant genera in the dorsal epithelium fraction were *Campylobacter* (26.9 ± 4.6%), followed by *Rikenellaceae* RC9 (6.5 ± 1.9%) and *Prevotella* (5.3 ± 1.6%).

### 3.3 Comparison of microbiota in the four yak rumen fractions

**Figure 3:**
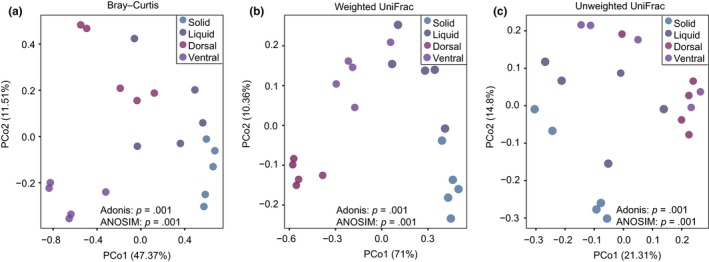
Principal coordinate analysis (PCoA) of microbial communities in the solid, liquid, dorsal epithelium, and ventral epithelium fractions of yak rumen based on (a) Bray–Curtis distance, (b) weighted uniFrac distance, and (c) unweighted uniFrac distance

To compare the microbial community among the different rumen fractions, the PCoA based on the Bray–Curtis distance, unweighted UniFrac distance, and weighted UniFrac distance was employed (Figure 3). The results showed that the bacterial communities clearly separated according to the rumen fractions. Moreover, the microbiota in the dorsal epithelium also distinguished from that in the ventral epithelium. Adonis analysis and ANOSIM based on the three distance matrices also revealed significant differences in microbial communities of the four fractions (*p* = .001, Figure 3).

**Figure 4:**
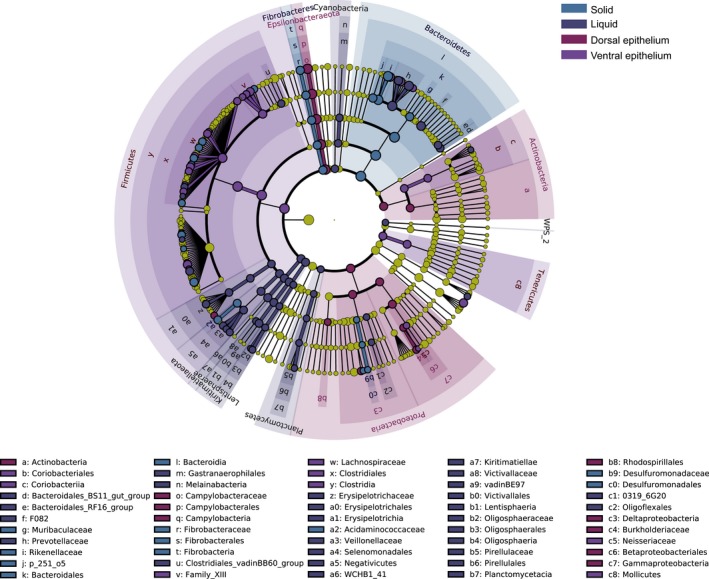
LEfSe cladogram showing the taxonomic differences among the four fractions in the rumen of yak. All identified taxonomy was significantly different based on Kruskal–Wallis test (*p* < .05) and a LDA score larger than 3.0

Application of LEfSe identified 120 bacterial taxa that were significantly enriched in the fractions (Figure 4): 27, 49, 16, and 28 in samples of the solid, liquid, dorsal epithelium, and ventral epithelium fractions, respectively. In the solid fraction, 13 genera were identified with relative abundance greater than 0.1%: *Rikenellaceae* RC9, *Fibrobacter*, *Anaerovorax*, *Lachnoclostridium* 10, *Lachnospiraceae* FCS020, *Lachnospiraceae* NK4A136, *Lachnospiraceae* UCG 006, *Mailhella*, *Papillibacter*, *Pelobacter*, *Ruminiclostridium* 6, *Ruminococcus* 1, and *Succiniclasticum*. In the liquid fraction, 11 genera met this criterion: *Bacteroidales* RF16, *Prevotella*, *Prevotellaceae* UCG 003, *Roseburia*, *Ruminococcus gauvreauii*, *Ruminococcaceae* UCG 010, *Erysipelotrichaceae* UCG 004, *Quinella*, *Selenomonas* 1, *Veillonellaceae* UCG 001, and *Fretibacterium*. In the dorsal epithelium fraction, four enriched genera were identified: *Comamonas*, *Campylobacter*, *Desulfovibrio,* and *Solobacterium*. Finally, in the ventral epithelium fraction, 13 genera with relative abundance >0.1% were identified: *Treponema* 2, *Howardella*, *Lachnospiraceae* UCG 010, *Lachnospiraceae* UCG 008, *Lachnospiraceae* NK3A20, *Acetitomaculum*, *Mogibacterium*, *Alloprevotella*, *Syntrophococcus*, *Ruminococcaceae* UCG 005, *Ruminococcaceae* UCG 009, *Prevotellaceae* UCG 001, and *Eubacterium nodatum* (Table 1).

**Table 1:** Relative abundances of bacterial taxa identified by LEfSe in microbiota of the solid, liquid, dorsal, and ventral epithelium fractions of yak rumen

Fraction	Enriched taxa	Relative abundance (Mean ± *SD*)			
		Solid	Liquid	Dorsal Ep	Ventral Ep
Solid	*Anaerovorax*	0.51 ± 0.09	0.44 ± 0.20	0.32 ± 0.11	0.19 ± 0.04
	*Fibrobacter*	3.48 ± 0.62	1.14 ± 0.60	2.60 ± 1.33	1.92 ± 0.82
	*Lachnoclostridium* 10	0.80 ± 0.35	0.29 ± 0.22	0.23 ± 0.19	0.22 ± 0.12
	*Lachnospiraceae* FCS020	0.63 ± 0.09	0.21 ± 0.06	0.22 ± 0.13	0.20 ± 0.11
	*Lachnospiraceae *NK4A136	0.83 ± 0.26	0.64 ± 0.20	0.48 ± 0.26	0.31 ± 0.14
	*Lachnospiraceae* UCG 006	0.30 ± 0.03	0.16 ± 0.10	0.14 ± 0.10	0.07 ± 0.04
	*Mailhella*	0.10 ± 0.07	0.06 ± 0.02	0.05 ± 0.02	0.03 ± 0.02
	*Papillibacter*	1.15 ± 0.43	0.64 ± 0.20	0.46 ± 0.25	0.41 ± 0.09
	*Pelobacter*	0.11 ± 0.02	0.03 ± 0.01	0.04 ± 0.02	0.02 ± 0.01
	*probable genus* 10	0.40 ± 0.13	0.22 ± 0.07	0.19 ± 0.12	0.21 ± 0.06
	*Rikenellaceae* RC9	22.55 ± 3.52	14.52 ± 4.64	8.26 ± 2.74	6.47 ± 1.91
	*Ruminiclostridium* 6	0.05 ± 0.04	0.04 ± 0.01	0.02 ± 0.02	0.01 ± 0.01
	*Ruminococcus* 1	3.57 ± 0.70	1.31 ± 0.62	1.00 ± 0.40	1.08 ± 0.73
	*Succiniclasticum*	1.57 ± 0.77	0.73 ± 0.30	0.41 ± 0.17	0.19 ± 0.08
Liquid	*Eubacterium oxidoreducens*	0.02 ± 0.01	0.01 ± 0.01	0.02 ± 0.01	0.03 ± 0.02
	*Ruminococcus gauvreauii*	0.05 ± 0.04	0.35 ± 0.21	0.09 ± 0.02	0.07 ± 0.03
	*Bacteroidales* RF16	0.23 ± 0.03	1.09 ± 0.51	0.98 ± 0.37	0.65 ± 0.34
	*Enterorhabdus*	0.01 ± 0.00	0.05 ± 0.03	0.01 ± 0.01	0.01 ± 0.01
	*Erysipelotrichaceae* UCG 004	0.25 ± 0.08	0.91 ± 0.50	0.76 ± 0.24	0.44 ± 0.15
	*Fretibacterium*	0.04 ± 0.01	0.17 ± 0.07	0.14 ± 0.07	0.11 ± 0.08
	*horsej‐a03*	0.14 ± 0.06	0.25 ± 0.03	0.14 ± 0.04	0.10 ± 0.05
	*Oscillospira*	0.01 ± 0.01	0.04 ± 0.03	0.02 ± 0.02	0.00 ± 0.00
	*p−1088‐a5 gut group*	0.09 ± 0.05	0.15 ± 0.08	0.03 ± 0.02	0.01 ± 0.01
	*Prevotella*	12.82 ± 2.23	14.59 ± 2.35	8.62 ± 3.03	5.26 ± 1.59
	*Prevotellaceae* UCG 003	2.61 ± 0.47	3.82 ± 0.47	1.61 ± 0.43	1.08 ± 0.22
	*Pseudobacteroides*	0.00 ± 0.00	0.03 ± 0.02	0.02 ± 0.01	0.01 ± 0.01
	*Quinella*	0.07 ± 0.06	0.68 ± 0.41	0.13 ± 0.09	0.04 ± 0.01
	*Roseburia*	0.02 ± 0.01	0.1 ± 0.04	0.09 ± 0.03	0.09 ± 0.04
	*Ruminococcaceae* UCG 010	1.15 ± 0.27	1.98 ± 0.53	1.06 ± 0.37	0.85 ± 0.30
	*Selenomonas* 1	0.06 ± 0.02	0.21 ± 0.09	0.03 ± 0.02	0.04 ± 0.02
	*Tyzzerella* 3	0.01 ± 0.01	0.02 ± 0.01	0.01 ± 0.01	0.01 ± 0.01
	*Veillonellaceae* UCG 001	0.32 ± 0.08	0.37 ± 0.15	0.08 ± 0.04	0.08 ± 0.03
Dorsal epithelium	*Campylobacter*	0.01 ± 0.01	3.13 ± 5.64	4.95 ± 2.4	26.91 ± 4.55
	*Comamonas*	0.00 ± 0.00	0.16 ± 0.22	0.31 ± 0.35	0.50 ± 0.29
	*Desulfovibrio*	0.08 ± 0.03	0.22 ± 0.03	0.38 ± 0.17	0.62 ± 0.65
	*Solobacterium*	0.01 ± 0.01	0.06 ± 0.09	0.08 ± 0.08	0.26 ± 0.18
Ventral epithelium	*Eubacterium nodatum*	0.02 ± 0.01	0.25 ± 0.23	2.26 ± 1.24	1.92 ± 0.92
	*Acetitomaculum*	0.05 ± 0.02	0.16 ± 0.06	0.57 ± 0.62	0.31 ± 0.29
	*Alloprevotella*	0.00 ± 0.01	0.08 ± 0.13	0.35 ± 0.36	0.27 ± 0.31
	*Blvii28 wastewater‐sludge* group	0.00 ± 0.00	0.51 ± 1.08	5.50 ± 4.82	1.90 ± 1.05
	*Family XIII AD3011*	0.07 ± 0.03	0.18 ± 0.11	0.48 ± 0.25	0.38 ± 0.17
	*GCA−900066575*	0.00 ± 0.00	0.02 ± 0.04	0.14 ± 0.23	0.02 ± 0.02
	*Howardella*	0.00 ± 0.00	0.08 ± 0.11	0.99 ± 0.86	0.79 ± 0.82
	*Lachnospiraceae* NK3A20	0.15 ± 0.06	0.21 ± 0.10	0.6 ± 0.32	0.34 ± 0.14
	*Lachnospiraceae* UCG008	0.50 ± 0.17	0.34 ± 0.13	1.47 ± 0.97	0.81 ± 0.18
	*Lachnospiraceae* UCG 010	0.01 ± 0.01	0.09 ± 0.18	0.4 ± 0.47	0.16 ± 0.09
	*Mogibacterium*	0.01 ± 0.00	0.03 ± 0.02	0.21 ± 0.17	0.11 ± 0.04
	*Prevotellaceae* UCG 001	2.65 ± 0.63	3.46 ± 1.15	5.58 ± 1.63	4.05 ± 1.16
	*Ruminococcaceae* UCG 004	0.01 ± 0.01	0.02 ± 0.01	0.04 ± 0.02	0.04 ± 0.03
	*Ruminococcaceae* UCG 005	0.42 ± 0.23	0.77 ± 0.63	3.85 ± 2.5	2.65 ± 1.21
	*Ruminococcaceae* UCG 009	0.02 ± 0.01	0.03 ± 0.02	0.1 ± 0.15	0.09 ± 0.04
	*Sutterella*	0.01 ± 0.01	0.02 ± 0.01	0.02 ± 0.01	0.00 ± 0.00
	*Syntrophococcus*	0.02 ± 0.01	0.05 ± 0.03	0.41 ± 0.41	0.16 ± 0.11
	*Treponema* 2	3.62 ± 1.09	1.13 ± 1.42	3.72 ± 1.92	2.32 ± 1.51

Abbreviation: Ep, epithelium.

### 3.4 qPCR quantification of bacterial density

The real‐time PCR results showed that the bacterial 16S rRNA gene copy numbers in the solid and liquid fractions were significantly higher than that in the dorsal and ventral epithelium fractions (*p* < .001, Figure 7). However, the differences between the solid and liquid fractions, and between the dorsal and ventral epithelium fractions were not significant (*p* > .05). In addition, the bacterial count in the ventral epithelium increased in comparison with that in the dorsal epithelium.

### 3.5 Potential functional profiles of yak rumen fractions

**Figure 5:**
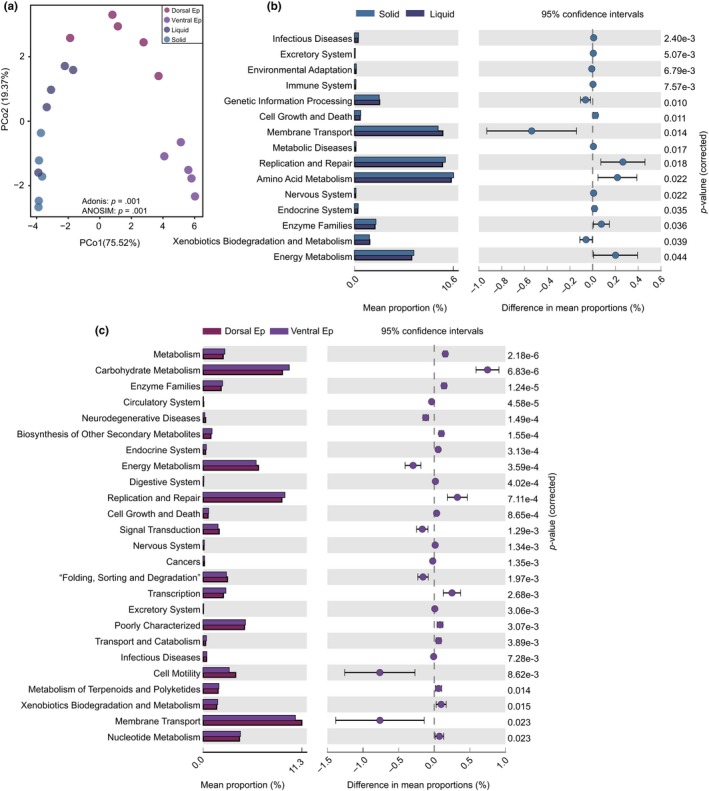
Functional profiles of microbial communities in the solid, liquid, and epithelial fractions. (a) PCoA plot revealing differences in predicted microbial functions based on Bray–Curtis distances. (b) Relative abundance of metabolic pathways in solid (green) and liquid (blue) fractions. The extended error bars show significantly different KEGG pathways between the fractions. (c) Relative abundances of pathways in the dorsal epithelium (orange) and ventral epithelium (maroon) fractions of yak. The extended error bars show significantly different KEGG pathways between the fractions. Ep, epithelium

To assess metabolic profiles of the ruminal microbiota, PICRUSt was applied to predict their functions (Figure 5). PCoA results showed that the functional profiles of the four fractions significantly differed (Figure 5a). Comparison of enriched KEGG pathways between the solid and liquid fractions indicated that amino acid metabolism, enzyme families, and energy metabolism pathways were enriched in the solid fraction, relative to the liquid fraction, while xenobiotic biodegradation and metabolism pathways were enhanced in the liquid fraction (Figure 5b). Further comparison revealed that energy metabolism, cell motility, and membrane transport were enriched in the dorsal epithelium fraction relative to the ventral epithelium (Figure 5c), while carbohydrate metabolism, enzyme families, metabolism of terpenoids and polyketides, and biodegradation of xenobiotics were stronger in the dorsal fraction than the ventral epithelium fraction (Figure 5c).

### 3.6 Comparison of ventral epithelium microbiota in yak and cattle

**Figure 6:**
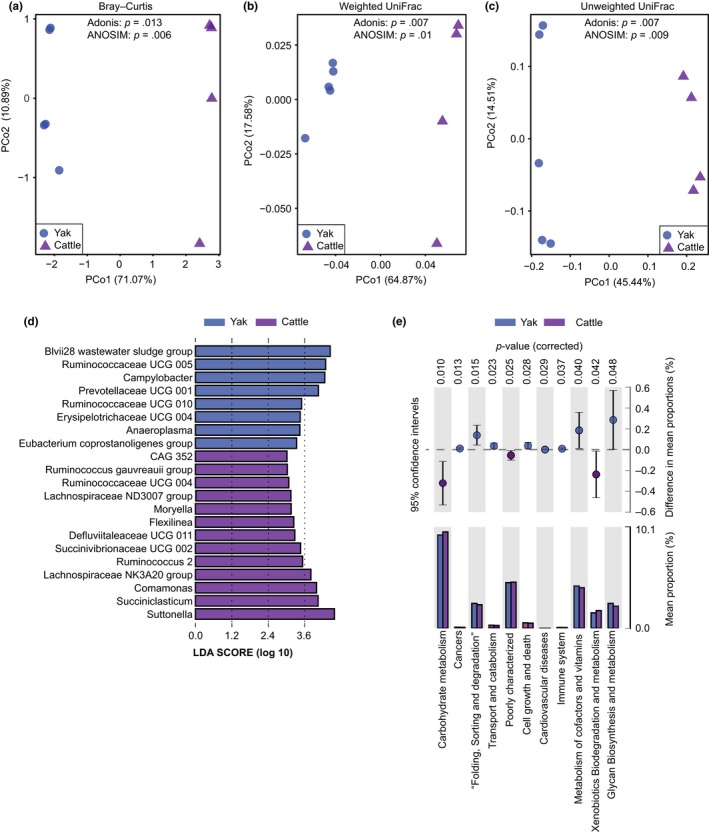
Differences in bacterial communities associated with the ventral epithelium between yak and cattle. Results of PCoA showing differences in the communities based on: (a) Bray–Curtis distance, (b) weighted uniFrac distance, and (c) unweighted uniFrac distance. (d) Results of LEfSe analysis showing taxa that significantly differed in the ventral epithelium of cattle and yak. (e) Differences in predicted functions between the ventral epithelium fraction of yak (orange) and cattle (purple). The extended error bars show significantly different KEGG pathways between the two ruminants’ ventral epithelium fractions

PCoA based on Bray–Curtis, unweighted uniFrac, and weighted uniFrac distances, to further elucidate roles of microbiota in the ventral epithelium of yak rumen, showed that they significantly differed from corresponding microbiota in cattle (Figure 6a‐c). These results were corroborated by ANOSIM (*p* < .05) and Adonis analysis (*p < *.01).

Application of LEfSe to explore the differences in microbial composition identified 20 genera as biomarkers distinguishing yak and cattle microbiota (Figure 6d). In the ventral epithelium fraction of yak, a total of seven taxa with relative abundance greater than 0.1% were identified (Table A1 in Appendix): *Campylobacter*, *Ruminococcaceae* UCG 005, *Ruminococcaceae* UCG 010, *Prevotellaceae* UCG 001, *Erysipelotrichaceae* UCG 004, *Anaeroplasma,* and *Eubacterium coprostanoligenes* group. In the ventral fraction of cattle, 13 genera were found to be enriched with relative abundance >0.1%: CAG 352, *Ruminococcus gauvreauii* group, *Ruminococcaceae* UCG 004, *Lachnospiraceae* ND3007 group, *Moryella*, *Flexiinea*, *Defluviitaleaceae* UCG 002, *Succinivibrionaceae* UCG 002, *Ruminococcus* 2, *Lachnospiraceae* NK3A30 group, *Comamonas*, *Succiniclasticum,* and *Suttonella*.

In addition, comparison of KEGG pathways enriched in the ventral epithelium fractions of yak and cattle revealed that cell growth and death, immune system, metabolism of cofactors and vitamins, and glycan biosynthesis and metabolism pathways were stronger in the yak fraction than the cattle fraction, while carbohydrate metabolism and xenobiotic biodegradation pathways were enhanced in the cattle fraction.

## 4 DISCUSSION

Our examination of microbiota in the yak rumen revealed clear differences in microbial composition and functional profiles among the solid, liquid, and dorsal and ventral epithelium fractions. The results also revealed clear differences in microbiota in the ventral epithelium fractions of yak and cattle. Our results extend understanding of the composition and roles of rumen microbiota in yak living on the QTP, as summarized in the following sections.

**Figure 7:**
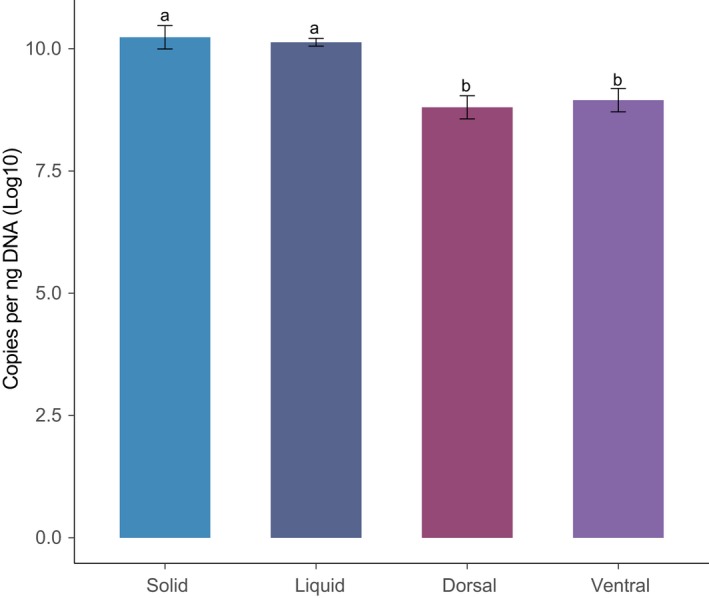
Density of bacteria in the solid, liquid, and epithelial fractions of yak rumen based on 16S rRNA gene. The results were expressed as log10 gene copies per ng of DNA. The different letters denote significant differences

Our results demonstrated that the bacteria density (Figure 7) and Shannon index of bacterial diversity were lower in the ventral epithelium fraction than in the solid and liquid fractions (Figure 1), in accordance with patterns previously detected in dairy cattle (De Mulder et al., 2017; Schären et al., 2017). Interestingly, we found that ventral epithelium had a much higher Shannon index and bacteria count than the dorsal epithelium, providing the first indications that the microbial community associated with the ventral epithelium is much more diverse.

We also found that members of the phyla *Bacteroidetes* and *Firmicutes* were the dominant bacteria in the solid, liquid, and ventral epithelium fractions of yak rumen, in accordance with previous findings in ruminants generally (Henderson et al., 2015) and yaks specifically (Hu et al., 2019; Xue et al., 2018; Zhang et al., 2016; Zhou, Zhong, et al., 2017a; Zhou, Fang, et al., 2017b). These results indicate that the two phyla have adapted to wide ranges of gastrointestinal tract environments and play important roles in rumen ecology. However, *Epsilonbacteraeota* was the most abundant bacterial phylum in the yak dorsal epithelium fraction, which has not been recorded in previous studies (An, Dong, & Dong, 2005; Guo et al., 2015; Hu et al., 2019; Xue et al., 2018; Zhang et al., 2016; Zhou, Zhong, et al., 2017a; Zhou, Fang, et al., 2017b). The comparative genomic analysis revealed that the host‐associated *Epsilonbacteraeota* lacked genes involved in carbon fixation, but possessed genes involved in osmoprotection, and transport of heme, lipopolysaccharide, and capsular polysaccharides (Waite et al., 2017), in accordance with presumed requirements for their epithelium‐associated lifestyle. These findings confirm, *inter alia*, the strength of effects of ecological niches on microbial communities.

We also found that *Rikenellaceae* RC9 and *Prevotella* were abundant genera in all four fractions, in accordance with previous findings regarding ruminants generally (Henderson et al., 2015) and yaks (Hu et al., 2019; Xue et al., 2018; Zhang et al., 2016). *Rikenellaceae* RC9 can reportedly degrade structural carbohydrates and starch in the rumen of cows (Asma et al., 2013), while *Prevotella* spp. have high genetic and metabolic diversity (Bekele, Koike, & Kobayashi, 2010; Purushe et al., 2010), and play key roles in metabolism of carbohydrates, such as hemicellulose, starch, xylan, and pectin (Cotta, 1992; Dehority, 1966; Gardner, Wells, Russell, & Wilson, 1995; Kabel et al., 2011), and nitrogen (Kim et al., 2017; Stevenson & Weimer, 2007). Thus, these results indicate that *Rikenellaceae* RC9 and *Prevotella* play crucial roles in basic metabolic processes in the yak rumen.

Our study also revealed significant differences among the microbial communities of the four fractions (Figure 4 and Table 1). The families *Lachnospiraceae* and *Ruminococcaceae*, *Succiniclasticum* spp., and *Fibrobacter* spp. were enriched in the solid fractions. Previous studies have shown that *Lachnospiraceae* and *Ruminococcaceae* are also relatively abundant in the solid phase of cattle rumen (De Mulder et al., 2017; Schären et al., 2017) and play important roles in degradation of cellulose and fermentation of plant fibers (Biddle, Stewart, Blanchard, & Leschine, 2013; Flint & Louis, 2009; Schwarz, 2001). Moreover, *Ruminococcaceae* are reportedly associated with feed efficiency of dairy cattle (Myer, Wells, Smith, Kuehn, & Freetly, 2015), *Fibrobacter* spp. are reportedly major degraders of cellulosic plant biomass in the herbivore gut (Ransom‐Jones, Jones, McCarthy, & McDonald, 2012), and *Succiniclasticum* spp. specialize in fermentation of succinate, yielding propionate as a major product (van Gylswyk, 1995). Therefore, these bacteria likely contribute strongly to digestion of fibers in the yak rumen, a hypothesis supported by our predictions of microbial functions (Figure 5).

The liquid fraction was enriched with *Roseburia* spp., *Quinella* spp., *Fretibacterium* spp., *Ruminococcus gauvreauii*, *Erysipelotrichaceae* UCG 004, and *Selenomonas* 1 (Figure 4 and Table 1). *Roseburia spp.* are important butyrate‐producing bacteria, which widely degrade starch through production of extracellular amylase (Duncan et al., 2006). *Ruminococcus gauvreauii* is also a glucose‐fermenting bacterium, mainly producing acetate (Domingo et al., 2008). *Quinella* spp. are reportedly associated with low methane production rates in sheep, and ferment sugars equimolarly to acetate and propionate (Krumholz et al., 1993). Thus, their abundance in the yak rumen is consistent with yaks’ low methane emissions (Zhang et al., 2016). *Selenomonas* spp. are obligately saccharolytic bacteria that participate in fermentation of soluble sugars and lactate in the rumen (Hespell, Paster, & Dewhirst, 2006). Interestingly, two species of the genus were previously isolated from yak rumen and shown to generate acetate and propionate through glucose fermentation (Zhang & Dong, 2009). Moreover, amino acid metabolism is enhanced in the liquid fraction relative to the solid fraction (Figure 5). Taken together, these results suggest that the enriched bacteria in the liquid fraction are important for the metabolism of soluble nutrients in the yak rumen.

Our results also revealed differences in the microbial composition and predicted functions between the dorsal and ventral epithelium (Figure 4 and Table 1). *Campylobacter* spp., *Desulfovibrio* spp., and *Comamonas* spp. were enriched in the dorsal epithelium, while *Treponema* 2, *Howardella* spp., members of the families *Lachnospiraceae* and *Ruminococcaceae*, *Acetitomaculum* spp., *Alloprevotella* spp., and *Syntrophococcus* spp. were identified in the ventral epithelium fraction. These findings are consistent with previous reports regarding microbiota in the ventral epithelium of cows (De Mulder et al., 2017; Liu et al., 2016; Mann, Wetzels, Wagner, Zebeli, & Schmitz‐Esser, 2018). *Campylobacter* spp. are microaerophilic bacteria that can consume oxygen, are positively correlated with the weight and papilla length of goat rumen (Jiao, Huang, Zhou, & Tan, 2015), and reportedly associated with nitrogen metabolism (Mann et al., 2018). *Comamonas* spp. are aerobic proteobacteria (Willems & De Vos, 2006), and thus are likely involved in oxygen scavenging. *Desulfovibrio* spp. can use oxygen as an electron acceptor under microaerophilic conditions and are involved in hydrogen metabolism (Voordouw, 1995). These results suggest that major activities of the microbiota in the dorsal epithelium likely involved in hydrogen metabolism and oxygen scavenging, in accordance with the predicted microbial functions (Figure 5).

Finally, we found significant differences between the ventral epithelium microbiota of yak and cattle, including enrichment of *Ruminococcaceae* UCG 005, *Anaeroplasma,* and *Erysipelotrichaceae* UCG 004 in yak, and enrichment of *Succiniclasticum*, *Ruminococcus* 2, and *Lachnospiraceae* NK3A20 in cattle (Figure 6 and Table A1 in Appendix). The representative OTU sequences of *Ruminococcaceae* UCG 005 are similar to those of *Sporobacter termitidis*, a hydrogen‐consuming acetogen isolated from the termite gut (91%–92% sequence similarity, according to BLAST analysis; Grech‐Mora et al., 1996). Members of *Anaeroplasma* reportedly induce increases in levels of mucosal IgA (Beller et al., 2019). *Erysipelotrichaceae* UCG 004 belongs to the family *Erysipelotrichaceae*, which is reportedly associated with inflammation‐related gastrointestinal diseases (Chen, Liu, Ling, Tong, & Xiang, 2012). *Succiniclasticum* reportedly plays an important role in fermentation of succinate to propionate (van Gylswyk, 1995). *Ruminococcus* 2 has amylolytic activity (Ferrario et al., 2017), and *Lachnospiraceae* NK3A20 can potentially biohydrogenate fatty acids (Wang et al., 2017). These findings are consistent with the functional predictions (Figure 6e) and indicate that microbiota associated with yak ventral epithelium may contribute more strongly to immune responses than the corresponding microbiota in cattle, which may have mainly metabolic roles. However, rumen microbiota are also significantly affected by dietary composition (Henderson et al., 2015). Therefore, the significant difference in the ventral epithelium microbiota between cattle and yak is likely to result from the dietary difference. In further study, it is necessary to compare rumen microbiota between cattle and yak fed the same diet.

## 5 CONCLUSION

In this study, we characterized and compared the microbiota in the solid, liquid, and epithelial (dorsal and ventral) fractions of yak rumen. The results show that the rumen microbiota significantly differ in the four ecological niches, in both composition and functions. They also provide the first information on microbial community in the dorsal epithelial fraction, which clearly differed from the community in the ventral epithelium. The predicted functional profiles showed that amino acid metabolism, enzyme families, and energy metabolism pathways were enriched in the solid fraction, while xenobiotic biodegradation and metabolic pathways were enriched in the liquid fraction. The results also indicate that microbiota of the ventral epithelium of yak and cattle substantially differ. Overall, the results extend knowledge of microbiota in the yak rumen and their roles in yaks’ adaptation to their harsh environments.

## ETHICS STATEMENT

All procedures applied were approved by the Institutional Animal Care and Use Committee of Lanzhou University and were in accordance with the university's guidelines for animal research.

## CONFLICT OF INTEREST

None declared.

## AUTHOR CONTRIBUTIONS

QMR and HZS collected the samples and analyzed the data; QMR, HZS, XTY, and CL analyzed the data; QMR, ZPL, and QQ wrote the manuscript; LMD and RJL participated in methodology and investigation; ZPL and QQ designed the study and reviewed the manuscript. All authors approved the final manuscript.

## Data Availability

The datasets generated in this study are available in the NCBI Sequence Read Archive database (SRP212418) at https://www.ncbi.nlm.nih.gov/sra/?term=SRP212418

## Appendix 1

**Table A1:** Relative abundances of the taxa identified by LEfSe that significantly differed in abundance between ventral epithelium fractions of yak and cattle rumen [Correction added on 6 January 2020 after first online publication: Table A1 from Supporting Information has been moved to Appendix section]

Fraction	Taxa	Relative abundance (Mean ± SD)	
		Yak	Cattle
Yak	*Blvii28 wastewater‐sludge*	5.50±4.82	0.01±0.01
	*Ruminococcaceae*UCG005	3.85±2.50	0.17±0.04
	*Campylobacter*	4.95±2.40	0.87±0.21
	*Prevotellaceae* UCG 001	5.58±1.63	3.21±0.28
	*Ruminococcaceae*UCG010	1.06±0.37	0.48±0.08
	*Erysipelotrichaceae*UCG004	0.76±0.24	0.18±0.07
	*Anaeroplasma*	0.72±0.54	0.16±0.08
	*Eubacterium coprostanoligenes*	0.75±0.10	0.32±0.15
Cattle	*CAG‐352*	0.0±0.0	0.21±0.23
	*Ruminococcus gauvreauii*	0.09±0.02	0.32±0.26
	*Ruminococcaceae *UCG 004	0.04±0.02	0.28±0.14
	*Lachnospiraceae *ND3007	0.10±0.06	0.39±0.23
	*Moryella*	0.01±0.01	0.29±0.14
	*Flexilinea*	0.05±0.02	0.38±0.07
	*Defluviitaleaceae* UCG 011	0.07±0.05	0.42±0.20
	*Succinivibrionaceae* UCG 002	0.01±0.01	0.63±0.76
	*Ruminococcus* 2	0.03±0.03	0.73±0.42
	*Lachnospiraceae* NK3A20	0.60±0.32	1.86±0.71
	*Comamonas*	0.31±0.35	1.96±1.14
	*Succiniclasticum*	0.41±0.17	2.46±0.72
	*Suttonella*	0.08±0.11	6.15±6.24
